# Supplementary material for: A Comprehensive Assessment of Ultraviolet-Radiation-Induced Mutations in Flammulina filiformis Using Whole-Genome Resequencing
Source: J Fungi (Basel). 2024 Mar 20;10(3):228. doi: 10.3390/jof10030228 (PMC10971301; doi:10.3390/jof10030228)
Supplement: Supplementary file 1 [file jof-10-00228-s001.zip › Supplementary Material S8/KEGG annotation/out/64381550635650.os/KO/out_map/map04921.html]

KEGG PATHWAY: Oxytocin signaling pathway - Reference pathway


|  |  |
| --- | --- |
| **Oxytocin signaling pathway - Reference pathway** |  |

[
Pathway menu
| Organism menu
| Pathway entry
| Show description
| User data mapping
]

Oxytocin (OT) is a nonapeptide synthesized by the magno-cellular neurons located in the supraoptic (SON) and paraventricular (PVN) nuclei of the hypothalamus. It exerts a wide variety of central and peripheral effects. However, its best-known and most well-established roles are stimulation of uterine contractions during parturition and milk release during lactation. Oxytocin also influences cardiovascular regulation and various social behaviors. The actions of OT are all mediated by one type of OT receptor (OTR). This is a transmembrane receptor belonging to the G-protein-coupled receptor superfamily. The main signaling pathway is the Gq/PLC/Ins3 pathway, but the MAPK and the RhoA/Rho kinase pathways are also activated, contributing to increased prostaglandin production and direct contractile effect on myometrial cells. In the cardiovascular system, OTR is associated with the ANP-cGMP and NO-cGMP pathways, which reduce the force and rate of contraction and increase vasodilatation.

|  |  |
| --- | --- |
| Reference pathway | 100% |
